# Association between Occupational Dysfunction and Social Isolation in Japanese Older Adults: A Cross-Sectional Study

**DOI:** 10.3390/ijerph18126648

**Published:** 2021-06-21

**Authors:** Keisuke Fujii, Yuya Fujii, Yuta Kubo, Korin Tateoka, Jue Liu, Koki Nagata, Shuichi Wakayama, Tomohiro Okura

**Affiliations:** 1 Department of Occupational Therapy, Faculty of Health Sciences, Kansai University of Health Sciences, 2-11-1 Wakaba, Kumatori, Sennnan, Osaka 590-0482, Japan; 2 Physical Fitness Research Institute, Meiji Yasuda Life Foundation of Health and Welfare, 150 Tobuki, Hachioji, Tokyo 192-0001, Japan; yu3-fujii@my-zaidan.or.jp; 3 Division of Occupational Therapy, Faculty of Rehabilitation and Care, Seijoh University, 2-172 Fukinodai, Tokai 476-8588, Japan; kubo-yu@seijoh-u.ac.jp; 4 Doctoral Program in Physical Education, Health and Sport Sciences, Degree Programs in Comprehensive Human Sciences, Graduate School of Comprehensive Human Sciences, University of Tsukuba, 1-1-1 Tennodai, Tsukuba 305-8577, Ibaraki, Japan; s2130461@s.tsukuba.ac.jp (K.T.); s1830468@s.tsukuba.ac.jp (J.L.); 5 Doctoral Program in Public Health, Degree Programs in Comprehensive Human Sciences, Graduate School of Comprehensive Human Sciences, University of Tsukuba, 1-1-1 Tennodai, Tsukuba 305-8577, Ibaraki, Japan; s2030441@s.tsukuba.ac.jp; 6 Department of Occupational Therapy, Ibaraki Prefectural University of Health Sciences, 4669-2 Ami, Ami, Inashiki 300-0394, Ibaraki, Japan; wakayamas@ipu.ac.jp; 7 Faculty of Health and Sport Sciences, University of Tsukuba, 1-1-1 Tennodai, Tsukuba 305-8577, Ibaraki, Japan; okura.tomohiro.gp@u.tsukuba.ac.jp; 8 R&D Center for Tailor-Made QOL, University of Tsukuba, 1-1-1 Tennodai, Tsukuba 305-8577, Ibaraki, Japan

**Keywords:** occupational therapy, occupational function, social network, social isolation

## Abstract

We clarified the relationship between occupational dysfunction and social isolation among community-dwelling adults. We used a self-administered questionnaire with a cross-sectional study for 2879 independently living older adults in Kasama City, Japan. Participants responded to a self-reported questionnaire in November 2019. Occupational dysfunction and social isolation were assessed. The participants were classified into two groups: healthy occupational function group, and occupational dysfunction group. To examine the relationship between occupational dysfunction and social isolation, we performed a logistic regression analysis with social isolation as a dependent variable and occupational dysfunction as an independent variable. In the crude model, the occupational dysfunction group had a higher risk of social isolation than the healthy occupational function group (odds ratio (OR) = 2.04; 95% confidence interval (CI), 1.63–2.55; *p* < 0.001). In the adjusted model, the occupational dysfunction group had a higher risk of social isolation than the healthy occupational function group (OR = 1.51; 95% CI, 1.17–1.94; *p* = 0.001). The results showed that occupational dysfunction was significantly associated with social isolation. These results can be used in constructing a support method for social isolation from a new perspective.

## 1. Introduction

The world’s population is aging, and Japan has the highest aging rate worldwide. In Japan, as the older adult population has grown in proportion, the composition of households has undergone a change; among older adults, the number of “one-person households” and “households of only a couple” have increased [1]. Therefore, older adults are likely to face increasing social changes, including problems such as social isolation. Social isolation is defined as a state in which an individual lacks a sense of social belongingness, refrains from engaging with others, has a minimal number of social contacts, and displays a deficiency in fulfilling quality relationships [2]. According to a large cohort study of Japanese older adults, the rate of social isolation was reported to be 17.7%, which means that nearly one in five Japanese older adults is experiencing social isolation [3]. Social isolation has been identified as a risk factor for poor health and well-being [4], cognitive decline [5], and mortality [6,7]. Therefore, measures to combat social isolation are important.

Difficulties related to occupational tasks or daily activities are called occupational dysfunction [8]. Occupational dysfunction is recognized worldwide as a major health-related problem in the preventive occupational therapy field [9]. It is a negative experience related to daily life and workplace activities, and it includes occupational marginalization, occupational imbalance, occupational alienation, and occupational deprivation [10]. Occupational dysfunction has been associated with poor mental health [11] and poor health-related quality of life [12]. As mental health and health-related quality of life are associated with social isolation [13,14,15], occupational dysfunction may also be associated with social isolation. For example, negative subjective daily life performance experiences (occupational dysfunction) may cause people to leave the social community where they participate in occupational activities, which may eventually lead to social isolation. A literature review by Papageorgiou et al. found evidence to support a positive relationship between occupation, participation, and prevention of social isolation among community-dwelling older adults [16]. Therefore, occupational therapists may prevent social isolation by supporting older adults’ occupational participation. 

However, there are no reports that examined the relationship between occupational dysfunction and social isolation in community-dwelling older adults. The primary purpose of this study is to clarify the relationship between occupational dysfunction and social isolation in older adults living in community-dwellings. The secondary purpose of this study is to determine which occupational dysfunction types are associated with social isolation.

## 2. Materials and Methods

### 2.1. Participants and Data Collection

This cross-sectional study was conducted in Kasama City, Ibaraki Prefecture, Japan. Kasama is a rural agricultural area, categorized as a flatland agricultural region and intermediate agricultural region. As of 1 January, 2021, Kasama City’s population is 73,589 and the aging rate is 32.4% [17]. 

**Figure 1:**
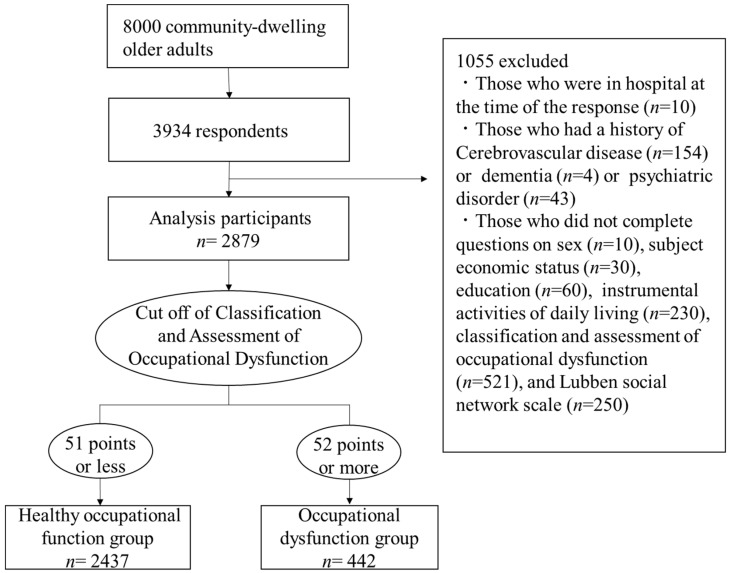
Participant flowchart.

Figure 1 shows the participant flowchart. Inclusion criteria were (1) those who are 65–85 years, (2) those without any functional disability. We randomly selected 8000 participants from the basic resident register on 1 October 2019. A self-administered questionnaire was mailed to participants in November 2019. Responses were obtained from 3934 persons (recovery rate: 49.2%). The exclusion criteria were as follows: (1) Those who were in hospital at the time of the response, (2) Those who had a history of cerebrovascular disease, dementia, or psychiatric disorder, and (3) Those who submitted incomplete questionnaires. As a result, 2879 participants were included in the final analysis. All participants were informed of the study details and provided informed consent. This study protocol was approved by the Ethical Committee of the University of Tsukuba (Ref No. Tai 019-101).

### 2.2. Measurement Variables

Demographic data including sex, age, household, educational background, subjective economic status, mental health, and instrumental activities of daily living (IADL) were used as covariates. Subjective economic status was assessed by the question, “How do you feel about your current economic situation?” Responses were rated on a scale ranging from “Very difficult,” “Slightly difficult,” “Normal,” “Somewhat rich,” “Very rich.” The two categories of “Very difficult” and “Slightly difficult” were operationally defined as “Poor.” Mental health status was assessed using the Japanese version of the Kessler 6 (K6) scale [18], a screening scale that can effectively measure psychological distress as per the Diagnostic and Statistical Manual of Mental Disorders (DSM-IV) [19]. Respondents answered six items on a 5-point Likert scale and responses on each item were transformed to scores ranging from 0 to 4 points. Total scores range from 0 to 24. A higher total score corresponds to a poorer mental health condition. IADL was evaluated using the five items of the Tokyo Metropolitan Institute of Gerontology Index of Competence (TMIG-IC), based on a subjective evaluation by respondents [20,21]. The TMIG-IC was developed to measure higher-level functional capacity among older adults living in the community and has been commonly used in Japan [22]. These five items of TMIG-IC were used to evaluate IADL ability. Higher values indicate good IADL ability (range: 0–5 points). In this study, IADL disability was defined as an IADL ability score of less than 5 [20,21].

Occupational dysfunction was evaluated using the Classification and Assessment of Occupational Dysfunction (CAOD) [10]. The CAOD measures occupational dysfunction with 16 items. The Cronbach’s alpha for this scale was 0.902 [10]. In our study, it was 0.914. Responses are rated on a seven-point Likert-type scale from “strongly agree” = 7 to “strongly disagree” = 1. It has a four-factor structure of occupational imbalance (4–28 points), occupational deprivation (3–21 points), occupational alienation (3–21 points), and occupational marginalization (6–42 points). Occupational marginalization is defined as a person not having the opportunity to engage in desired daily activities, such as when, “I have opinions but nobody hears them,” and “It is like being required to talk to a partner who is unpleasant” etc. [23]. Occupational imbalance is a loss of balance in engaging in daily activities, such as feeling, “I am so busy that my life’s rhythm is confused,” “There is no time to rest, and I am tired,” “Daily life is becoming very busy and increasingly exhausting,” and “My busy life has led to lack of sleep,” etc. [24]. Occupational alienation is defined as a situation when the inner needs of the individual concerning daily activities are not satisfied, such as “I feel my life has no meaning,” “There is no sense of accomplishment in daily life,” and “Daily life has become tedious,” etc. [25]. Occupational deprivation is a lack of opportunity for daily activities beyond the individual’s control such as, “There is no place where I can enjoy hobbies,” “There is no opportunity to carry out that what I consider important for its own sake,” and “I cannot enjoy my favorite activities,” etc. [26]. The cutoff value of the CAOD was 52 points, and the higher the score, the more likely it indicated an occupational function impairment. The item characteristics, structural validity, and internal consistency of the CAOD have been confirmed for university students, health care workers, community-dwelling older adults, people with mental disorders, and people with physical disabilities [8,10].

Social isolation was evaluated using the Japanese version of the Abbreviated Lubben Social Network Scale (LSNS) [27]. The LSNS-6 consists of six items, three related to the number of people in the family network and three related to the number of people in the friends and acquaintances network. The Cronbach’s alpha for this scale was 0.83 [27]. In our study, it was 0.872. Responses are rated on a six-point scale (range: 0–30 points). Social isolation was defined as an LSNS score of less than 12 points [27].

### 2.3. Statistical Analysis

According to the cutoff value of CAOD, the participants were divided into the “healthy occupational function group (CAOD score ≤ 51 points)” and “occupational dysfunction group (CAOD score ≥ 52 points)”. We calculated the means and standard deviations for continuous variables and frequencies and percentages for categorical variables. Student’s *t*-test and chi-square test were used to compare the characteristics of health occupational function and occupational dysfunction groups. To examine the relationship between occupational dysfunction and social isolation, we performed a logistic regression analysis with social isolation as a dependent variable and occupational dysfunction as an independent variable. We used two models in this study: a crude model and an adjusted model. The latter was adjusted for age, sex, household, educational background, subjective economic status, mental health, and IADL ability. These covariates were selected as potential confounders from previous studies. A logistic regression analysis was conducted to clarify the relationship between occupational dysfunction types and social isolation, and each occupational dysfunction type was entered as a dependent variable.

Analysis was performed using STATA/MP 16.0 (Stata Corp., College Station, TX, USA). In all analyses, a *p*-value of <0.05 was considered to indicate statistical significance.

## 3. Results

The comparison of characteristics between the healthy occupational function and the occupational dysfunction group is shown in Table 1. The number of people with occupational dysfunction was 442 (15.4%). The occupational dysfunction group was significantly less likely to have an education level beyond high school (*p* = 0.001), have significantly worse subjective economic status (*p* < 0.001), higher mental health scores (*p* < 0.001), higher CAOD scores (*p* < 0.001), and higher scores for the occupational dysfunction types (*p* < 0.001). Social isolation was significantly higher among individuals with evidence of occupational dysfunction (*p* < 0.001). 

Table 2 shows the association between occupational dysfunction and social isolation. In the crude model, the occupational dysfunction group had a higher risk of social isolation than those with healthy occupational function group (odds ratio (OR) = 2.04; 95% confidence interval (CI), 1.63–2.55; *p* < 0.001). In the adjusted model, the occupational dysfunction group had a higher risk of social isolation than the healthy occupational function group (OR = 1.51; 95% CI, 1.17–1.94; *p* = 0.001). Table 3 shows the association between the classification of occupational dysfunction and social isolation. In the crude model, occupational imbalance (OR = 1.10; 95% CI, 1.08–1.13; *p* < 0.001), occupational alienation (OR = 1.15; 95% CI, 1.12–1.78; *p* < 0.001), and occupational deprivation (OR = 1.07; 95% CI, 1.05–1.09; *p* < 0.001) were significantly correlated with social isolation. In contrast, in the adjusted model, occupational marginalization (OR = 0.93; 95% CI, 0.90–0.96; *p* < 0.001), occupational alienation (OR = 1.10; 95% CI, 1.06–1.13; *p* < 0.001), and occupational deprivation (OR = 1.04; 95% CI, 1.01–1.07; *p* = 0.003) were significantly correlated with social isolation, but occupational imbalance was not significant (OR = 1.03; 95% CI, 0.99–1.06; *p* = 0.134).

## 4. Discussion

The present study is the first to examine the relationship between occupational dysfunction and social isolation. Social isolation was found to be significantly associated with occupational dysfunction. Those with occupational dysfunction had a significantly higher rate of social isolation (31.5%) compared to those with healthy occupational function (18.4%). Furthermore, compared with those with healthy occupational function, the adjusted odds ratio for social isolation among those with occupational dysfunction was significantly higher at 1.51. The adjusted odds ratio confirmed that the relationship was independent, even after adjusting for the effect of mental health, which was strongly associated with social isolation. A person is expected to relate to society through occupational participation [28]. As a result, occupational dysfunction is associated with social isolation, such as through having fewer relationships with others in the work-place surroundings. This study had a cross-sectional design and therefore, it is difficult to make a strong statement about causality. However, in a previous study reviewing research on social isolation in the field of occupational therapy, the authors identified the paucity of research focusing on social isolation and called for studies on interventions to prevent social isolation in occupational therapy practice [29]; this study meets this need. The findings suggest that occupational dysfunction needs to be considered when occupational therapists think about the problem of social isolation of older adults in the community. The results of this study may provide occupational therapists and other professionals working in the community with a new perspective on social isolation.

Additionally, the present study examined the relationship between occupational dysfunction type and social isolation and revealed that occupational imbalance, occupational alienation, and occupational marginalization, but not occupational deprivation, were associated with social isolation. Occupational alienation and occupational marginalization had significantly higher odds ratios for social isolation. Hence, it is important to pay particularly careful attention to these two occupational dysfunction types. Therefore, when occupational therapists provide support for older adults in the community, they should not only know the occupation of the individual but also understand how they perceive the “internal needs of the individual” and “evaluation by others” regarding the occupation. 

In this study, occupational dysfunction was assessed using the CAOD, which detects conditions caused by overwork. Therefore, a state of poor occupational imbalance, as assessed by CAOD, is considered a busy life rhythm in daily activities. People identifying with this condition may maintain social relationships in their busy life. For example, it is believed a person communicates while working. Therefore, the odds ratio of social isolation was lower for those with a poor occupational imbalance status, which was considered an inversion of the odds ratio. However, a state of occupational imbalance has the potential to cause burnout. In a previous study focusing on medical staff, the relationship between occupational dysfunction and occupational stress was examined [11]. High occupational stress has also been reported to be associated with the incidence of cognitive dysfunction [30]. Therefore, it was necessary to keep in mind that, although occupational imbalance was not negatively related to social isolation, it may negatively affect other health conditions. 

The present study did not find any relationship between occupational deprivation and social isolation. This result may relate to potential factors not evaluated in this study. For example, if a place to perform an important occupation is not available in the area of residence, occupational deprivation may be attributed to geographical problems and is unlikely to relate to social isolation. In addition, occupational deprivation could also be influenced by issues affecting mobility, accessibility, and availability. It is necessary to further investigate these factors in the future.

The results of this study need to be interpreted with caution. For instance, if the total CAOD score classifies the person into the occupational dysfunction group, but the scores for occupational alienation and work alienation are low, the results may be difficult to interpret. In order to cope with such cases, it is necessary to conduct research to calculate cutoff values for each type of work dysfunction in the future. On the other hand, there are cases in which the total score of CAOD falls into the healthy occupational function group, but the scores of occupational alienation and occupational alienation are high. In such case, these individuals may be considered as those who are in the healthy occupational function group but are at high risk of social isolation. 

This study examines the relationship between occupational dysfunction and social isolation, but it has several limitations. First, the cross-sectional research design does not allow inference of causality. In the future, longitudinal studies should be conducted to examine whether occupational dysfunction causes social isolation. Second, although this study had a sufficient sample size, the final study population was approximately 40% of the total. Because there may be a selection bias between those who responded to the questionnaire and those who did not, it is necessary to increase the response rate in the future. In addition, it is necessary to investigate this issue in other countries and various regions in Japan. Third, this study was conducted using a questionnaire survey method, so the results are based on participants’ self-reports. Therefore, there is a possibility of overestimation and underestimation. Fourth, in this study, those with a history of cerebrovascular diseases, dementia, and depression were excluded. Additionally, other medical comorbidities, such as arthritis and cardiovascular diseases, were not taken into account. Future studies are advised to consider these medical conditions due to their possible influence on both occupational dysfunction and social isolation. Despite the above limitations, our findings contribute to the development of public health policies and plans that promote the research and practice of new evidence-based occupational therapy approaches that focus on occupational alienation and marginalization to combat social isolation of community-dwelling older adults.

Occupational therapists utilize occupational participation to assist and empower individuals and populations to attain and/or manage their own physical and psychological health, well-being, and participation [31,32]. In addition, occupational therapists facilitate healthy aging in community dwelling older adults by addressing and promoting their occupational needs [32]. Through the results of this study, occupational therapists who provide support for occupational dysfunction may be able to contribute to the social isolation of older people living in the community. In the future, in addition to conducting longitudinal studies, we need to work on (1) practical research on occupational therapy for the prevention of social isolation and (2) practical research on occupational therapy for the improvement of social isolation.

## 5. Conclusions

In the present study, the relationship between occupational dysfunction and social isolation was examined in a cross-sectional study. The results showed that occupational dysfunction was significantly associated with social isolation. Furthermore, as a result of examining the relationship between the occupational dysfunction type and social isolation, it was found that occupational imbalance, occupational alienation, and occupational marginalization were significantly associated with social isolation. These results can be used in constructing a support method for social isolation from a new perspective. Further research involving longitudinal studies is needed to investigate causality in detail. In addition, there is a need to conduct intervention studies to prevent social isolation. This study adds to the occupational therapy evidence base and supports the important role and future potential of occupation as a form of intervention to facilitate healthy aging. In particular, supporting healthy occupational participation may be a means of addressing social isolation.

## Figures and Tables

**Table 1:** Participants’ characteristics.

Characteristic	Healthy Occupational Function Group (*n* = 2437)	Occupational Dysfunction Group (*n* = 442)	*p*-Value
	%(*n*)	%(*n*)	
Age (years), Mean ± SD	72.6 ± 5.2	72.7 ± 5.6	0.795
Female, %(*n*)	49.3 (1202)	46.2 (204)	0.220
Household (living alone), %(*n*)	12.7 (310)	12.7 (56)	0.976
Educational background (≧high school), %(*n*)	84.1 (2030)	77.8 (339)	0.001
Subjective economic status (poor), %(*n*)	17.0 (413)	31.9 (553)	*p* < 0.001
IADL ability (disability), %(*n*)	7.4 (181)	7.9 (35)	0.718
K6 (score), Mean ± SD	2.8 ± 3.0	6.2 ± 4.0	*p* < 0.001
Social isolation, %(*n*)	18.4 (448)	31.5 (139)	*p* < 0.001
CAOD (score), Mean ± SD	30.0 ± 10.5	60.6 ± 7.1	*p* < 0.001
Occupational imbalance (score), Mean ± SD	7.5 ± 3.8	14.8 ± 3.8	*p* < 0.001
Occupational deprivation (score), Mean ± SD	6.2 ± 3.2	12.8 ± 2.7	*p* < 0.001
Occupational alienation (score), Mean ± SD	6.4 ± 3.4	12.1 ± 2.8	*p* < 0.001
Occupational marginalization (score), Mean ± SD	9.9 ± 3.8	20.8 ± 3.8	*p* < 0.001

SD: standard deviation; IADL: instrumental activities of daily living; CAOD: classification and assessment of occupational dysfunction.

**Table 2:** Association between occupational dysfunction and social isolation.

	Crude Model			Adjusted Model		
	OR	95%CI	*p*-Value	OR	95%CI	*p*-Value
Healthy occupational function group	Ref.			Ref.		
Occupational dysfunction group	2.04	1.63–2.55	*p* < 0.001	1.51	1.17–1.94	0.001

OR: odds ratio, CI: confidence interval, Ref: reference. Adjusted model was adjusted for age, sex, household, educational background, subjective economic status, instrumental activities of daily living, and mental health.

**Table 3:** Association classification in the relationship between occupational dysfunction and social isolation.

Occupational Dysfunction Type	Crude Model			Adjusted Model		
	OR	95%CI	*p*-Value	OR	95%CI	*p*-Value
Occupational imbalance	1.01	0.99–1.03	0.248	0.93	0.90–0.96	*p* < 0.001
Occupational deprivation	1.10	1.08–1.13	*p* < 0.001	1.03	0.99–1.06	0.134
Occupational alienation	1.15	1.12–1.78	*p* < 0.001	1.10	1.06–1.13	*p* < 0.001
Occupational marginalization	1.07	1.05–1.09	*p* < 0.001	1.04	1.01–1.07	0.003

OR: odds ratio, CI: confidence interval. Adjusted model was adjusted for age, sex, household, educational background, subjective economic status, instrumental activities of daily living, mental health, and other types of occupational dysfunction.

## Data Availability

The data that support the findings of this study are available on request from the corresponding author. The data are not publicly available due to privacy or ethical restrictions.

## References

[1] Ministry of Health, Labour and Welfare (2019). Summary Report of Comprehensive Survey of Living Conditions 2019. https://www.mhlw.go.jp/english/database/db-hss/dl/report_gaikyo_2019.pdf.

[2] Nicholson N.R. (2009). Social isolation in older adults: An evolutionary concept Analysis. J. Adv. Nurs..

[3] Saito M., Kondo K., Ojima T., Hirai H., The JAGES Group (2015). Criteria for social isolation based on associations with health indicators among older people. A 10-year follow-up of the Aichi Gerontological Evaluation Study. Jpn. J. Public Health.

[4] Courtin E., Knapp M. (2017). Social isolation, loneliness and health in old age: A scoping review. Health Soc. Care Community.

[5] Kuiper J.S., Zuidersma M., Oude Voshaar R.C., Zuidema S.U., van den Heuvel E.R., Stolk R.P., Smidt N. (2015). Social relationships and risk of dementia: A systematic review and meta-analysis of longitudinal cohort studies. Ageing Res. Rev..

[6] Holt-Lunstad J., Smith T.B., Baker M., Harris T., Stephenson D. (2015). Loneliness and social isolation as risk factors for mortality: A meta-analytic review. Perspect. Psychol. Sci..

[7] Steptoe A., Shankar A., Demakakos P., Wardle J. (2013). Social isolation, loneliness, and all-cause mortality in older men and women. Proc. Natl. Acad. Sci. USA.

[8] Miyake Y., Eguchi E., Ito H., Nakamura K., Ito T., Nagaoka K., Ogino N., Ogino K. (2018). Association between occupational dysfunction and metabolic syndrome in community-dwelling Japanese adults in a cross-sectional study: Ibara Study. Int. J. Environ. Res. Public Health.

[9] Kielhofner G., Braveman B., Baron K., Fisher G., Hammel J., Littleton M. (1999). The model of human occupation: Understanding the worker who is injured or disabled. Work.

[10] Teraoka M., Kyougoku M. (2015). Development of the final version of the Classification and Assessment of Occupational Dysfunction Scale. PLoS ONE.

[11] Townsend E., Wilcock A.A. (2004). Occupational justice and client-centred practice: A dialogue in progress. Can. J. Occup. Ther..

[12] Anaby D., Jarus T., Backman C.L., Zumbo B.D. (2010). The role of occupational characteristics and occupational imbalance in explaining well-being. Appl. Res. Qual. Life.

[13] Bryant W., Craik C., McKay E.A. (2004). Living in a glasshouse: Exploring occupational alienation. Can. J. Occup. Ther..

[14] Whiteford G. (2000). Occupational deprivation: Global challenge in the new millennium. Br. J. Occup. Ther..

[15] Teraoka M., Kyougoku M. (2015). Analysis of structural relationship among the occupational dysfunction on the psychological problem in healthcare workers: A study using structural equation modeling. PeerJ.

[16] Morohoshi N., Kyougoku M. (2019). Analysis of structural relationships among occupational challenge, occupational participation, occupational dysfunction, depression, and health-related qol in community dwelling elderly with physical disabilities. Jpn. Occup. Ther. Res..

[17] Ge L., Yap C.W., Ong R., Heng B.H. (2017). Social isolation, loneliness and their relationships with depressive symptoms: A population-based study. PLoS ONE.

[18] Hawton A., Green C., Dickens A.P., Richards S.H., Taylor R.S., Edwards R., Greaves C.J., Campbell J.L. (2011). The impact of social isolation on the health status and health-related quality of life of older people. Qual. Life Res..

[19] Matthews T., Danese A., Wertz J., Odgers C.L., Ambler A., Moffitt T.E., Arseneault L. (2016). Social isolation, loneliness and depression in young adulthood: A behavioural genetic analysis. Soc. Psychiatry Psychiatr. Epidemiol..

[20] Papageorgiou N., Marquis R., Dare J., Batten R. (2016). Occupational Therapy and Occupational Participation in Community Dwelling Older Adults: A Review of the Evidence. Phys. Occup. Ther. Geriatr..

[21] Government of Kasama City (2018). Statistical Information of Kasama City. https://www.city.kasama.lg.jp/data/doc/1547708244_doc_81_0.pdf.

[22] Furukawa T.A., Kawakami N., Saitoh M., Ono Y., Nakane Y., Nakamura Y., Tachimori H., Iwata N., Uda H., Nakane H. (2008). The performance of the Japanese version of the K6 and K10 in the World Mental Health Survey Japan. Int. J. Methods Psychiatr. Res..

[23] Kessler R.C., Andrews G., Colpe L.J., Hiripi E., Mroczek D.K., Normand S.L., Walters E.E., Zaslavsky A.M. (2002). Short screening scales to monitor population prevalences and trends in non-specific psychological distress. Psychol. Med..

[24] Tomioka K., Kurumatani N., Hosoi H. (2017). Association between social participation and 3-year change in instrumental activities of daily living in community-dwelling elderly adults. J. Am. Geriatr. Soc..

[25] Tomioka K., Kurumatani N., Saeki K. (2018). The differential effects of type and frequency of social participation on IADL declines of older people. PLoS ONE.

[26] Koyano W., Shibata H., Nakazato K., Haga H., Suyama Y. (1987). Measurement of competence in the elderly living at home: Development of an index of competence. Nippon Koshu Eisei Zasshi.

[27] Lubben J., Blozik E., Gillmann G., Iliffe S., von Renteln Kruse W., Beck J.C., Stuck A.E. (2006). Performance of an abbreviated version of the lubben social network scale among three european community-dwelling older adult populations. Gerontologist.

[28] Larsson-Lund M., Nyman A. (2017). Participation and occupation in occupational therapy models of practice: A discussion of possibilities and challenges. Scand. J. Occup. Ther..

[29] Collins T., Davys D., Martin R., Russell R., Kenney C. (2020). Occupational therapy, loneliness and social isolation: A thematic review of the literature. Int. J. Ther. Rehabil..

[30] Giorgi G., Lecca L.I., Leon-Perez J.M., Pignata S., Topa G., Mucci N. (2020). Emerging Issues in Occupational Disease: Mental Health in the Aging Working Population and Cognitive Impairment—A Narrative Review. Biomed. Res. Int..

[31] American Occupational Therapy Association [AOTA] (2014). Occupational therapy practice framework: Domain and process (3rd ed.). Am. J. Occup. Ther..

[32] Stav W.B., Hallenen T., Lane J., Arbesman M. (2012). Systematic review of occupational engagement and health outcomes among community-dwelling older adults. Am. J. Occup. Ther..

